# Discrimination of Basal Cell Carcinoma from Normal Skin Tissue Using High-Resolution Magic Angle Spinning ^1^H NMR Spectroscopy

**DOI:** 10.1371/journal.pone.0150328

**Published:** 2016-03-02

**Authors:** Je-Ho Mun, Heonho Lee, Dahye Yoon, Byung-Soo Kim, Moon-Bum Kim, Shukmann Kim

**Affiliations:** 1 Department of Dermatology, Seoul National University College of Medicine, Seoul, Korea; 2 Institute of Human-Environment Interface Biology, Seoul National University, Seoul, Korea; 3 Department of Chemistry, Center for Proteome Biophysics and Chemistry Institute for Functional Materials, Pusan National University, Busan, Korea; 4 Department of Dermatology, Pusan National University School of Medicine, Busan, Korea; University of Pittsburgh School of Medicine, UNITED STATES

## Abstract

High-resolution magic angle spinning nuclear magnetic resonance (HR-MAS NMR) spectroscopy is a useful tool for investigating the metabolism of various cancers. Basal cell carcinoma (BCC) is the most common skin cancer. However, to our knowledge, data on metabolic profiling of BCC have not been reported in the literature. The objective of the present study was to investigate the metabolic profiling of cutaneous BCC using HR-MAS ^1^H NMR spectroscopy. HR-MAS ^1^H NMR spectroscopy was used to analyze the metabolite profile and metabolite intensity of histopathologically confirmed BCC tissues and normal skin tissue (NST) samples. The metabolic intensity normalized to the total spectral intensities in BCC and NST was compared, and multivariate analysis was performed with orthogonal partial least-squares discriminant analysis (OPLS-DA). P values < 0.05 were considered statistically significant. Univariate analysis revealed 9 metabolites that showed statistically significant difference between BCC and NST. In multivariate analysis, the OPLS-DA models built with the HR-MAS NMR metabolic profiles revealed a clear separation of BCC from NST. The receiver operating characteristic curve generated from the results revealed an excellent discrimination of BCC from NST with an area under the curve (AUC) value of 0.961. The present study demonstrated that the metabolite profile and metabolite intensity differ between BCC and NST, and that HR-MAS ^1^H NMR spectroscopy can be a valuable tool in the diagnosis of BCC.

## Introduction

Basal cell carcinoma (BCC), first described by Jacob in 1827, is the most common malignant neoplasm in humans [1]. In Caucasian populations in North America, the incidence of BCC has increased more than 10% a year, leading to a 30% risk of developing BCC during a lifetime [2]. BCC has become an important public health problem and is a significant burden to the national health care service [3]. This increasing incidence is likely due to increased surveillance and a combination of multiple risk factors, which include increased sun exposure, ultraviolet or ionizing radiation, genetic defects, and immunosuppression. BCC arises from non-keratinizing cells in the basal layer of the epidermis. Although BCC grows slowly and rarely metastasizes, it can invade the surrounding tissues and cause local tissue destruction, functional impairment, and cosmetic disfiguration. Therefore, early diagnosis and treatment are crucial for a favorable prognosis.

Metabolomics describes the “quantitative measurement of time-related multiparametric metabolic responses of multicellular systems to pathophysiological stimuli or genetic modifications” [4]. Whereas genomics and proteomics focus on upstream gene and protein products, metabolomics is concerned with downstream outputs of global cellular networking [5]. Biomarkers of interest include metabolites that are intermediates and final products of metabolism. Some of these biomarkers include molecules associated with energy storage and utilization, precursors to proteins and carbohydrates, regulators of gene expression, and signaling molecules [5]. Among the various techniques used in metabolomics, high-resolution magic angle spinning (HR-MAS) ^1^H nuclear magnetic resonance (NMR) spectroscopy is increasingly used to investigate metabolic profiles. The advantages of ^1^H NMR spectroscopy include non-destructive analysis of samples, high reproducibility, minimal sample preparation processes, fast examination, and analysis of the entire sample in a single measurement [6]. Multivariate statistical methods and pattern recognition programs have been developed to handle the acquired data and search for discriminating features of biological sample sets [7]. The combination of NMR with pattern recognition methods has proven highly effective in identifying unknown metabolites that correlate with changes in genotype or phenotype [7].

The use of HR-MAS ^1^H NMR spectroscopy as a diagnostic tool to evaluate the metabolism of various cancers has recently been investigated. The technique has been employed to assist in the diagnosis and characterization of various cancers, including breast, lung, gastric, renal, colorectal, cervical, prostate, oral, and head and neck carcinomas [8–22]. However, to our knowledge, the metabolic profile of BCC has not been examined. Therefore, in the present study, we investigated the metabolic profile of BCC using ^1^H HR-MAS spectroscopy.

## Materials and Methods

We carried out this study with histopathologically confirmed cutaneous BCC and normal skin tissues (NSTs). All tissues were acquired from patients with skin cancers who underwent Mohs micrographic surgery at the dermatologic surgical clinic of Pusan National University Yangsan Hospital. During the first stage of Mohs micrographic surgery, the main tumor was carefully excised. After confirmation of complete tumor removal, the resultant surgical defect was repaired by reconstructive surgery. NST was acquired from dog ear repair or flap surgery when available. All tumor and NST tissues were divided into 2 sections. Part of each tissue was sent to the pathology department to confirm the histopathologic diagnosis of the samples, and the other part of the tissue was immediately snap-frozen in liquid nitrogen and stored at -80°C until NMR analysis.

This study was approved by the institutional review board of Pusan National University Yangsan Hospital, and written informed consent was obtained from every patient. The study was conducted in compliance with the principles of the Declaration of Helsinki.

### Sample collection and preparation

Tissues were immediately frozen and stored at −80°C until analysis. Twenty-five milligrams of each sample was weighed prior to NMR analysis. The weighed tissue was then transferred to a 4-mm nanotube with 25 μL deuterium oxide to provide field lock, and 2 mM TSP-d4 (3-(trimethylsilyl) propionic-2,2,3,3-d4 acid sodium salt) as a reference. The rotor was capped, and the spinning speed was monitored and recorded. Samples were prepared as rapidly as possible in order to prevent contamination or enzymatic decomposition.

### NMR spectroscopy

All spectra were acquired at 600.167 MHz using an Agilent spectrometer operating at ^1^H frequency and equipped with a 4-mm gHX NanoProbe. High-resolution ^1^H NMR metabolic profiling of the biopsied tissue samples was achieved using magic angle spinning (MAS) at 54.7° with respect to the direction of the magnetic field. All data were collected at a spinning rate of 2,000 Hz, and the spectra were checked between the water peak and side band, which coincides with the spin rate. Spectra were acquired using the presat-CPMG (Carr-Purcell-Meiboom-Gill) pulse sequence to suppress water and high molecular mass compounds. The acquisition time was 1.704 sec and relaxation delay time was 1 sec. In total, 128 scans were collected at a spectral width of 9615.4 Hz at 299.1 K. A total measurement time of 8 min 13 sec was required per sample. All data were Fourier-transformed and calibrated to TSP-d4 as 0.00 ppm using Chenomx NMR Suite 7.1 professional software (Chenomx Inc., Edmonton, Canada).

### Data analysis

All spectra were processed and assigned using Chenomx NMR Suite 7.1 professional and the Chenomx 600 MHz library database. The Chenomx NMR Suite is an integrated set of tools that allows for the identification and quantification of metabolites in an NMR spectra. The Chenomx reference libraries contain hundreds of fully searchable pH dependent compound models. Single peaks were confirmed by analysis of each spike, and the overlapped data were analyzed by a 2D correlation spectroscopy (COSY) NMR spectrum (S1 Fig). All data were converted to the frequency domain, corrected for phase and baseline, and then the TSP-d4 singlet peak was adjusted to 0.00 ppm. The target profiling method involved the confirmation of changes in a specific metabolite, followed by the comparison of data from the normal and cancer samples using Chenomx. The pattern recognition process required statistical analysis software. In this study, the SIMCA-P+ 12.0 software package (Umetrics, Umea, Sweden) was utilized to identify differences in the metabolite profile and metabolite intensity of normal and cancer data. The spectra were normalized to the total area and then binned within 0.001 ppm using Chenomx. The water region (4.6 ppm to 4.7 ppm) and the reference peak were excluded prior to analysis. The orthogonal partial least-squares discriminant analysis (OPLS-DA) was performed with Pareto scaling to differentiate between the cancer and normal group. The quality of the model was demonstrated by the cross-validation parameters *R*^*2*^ and *Q*^*2*^. The relative concentrations of marker metabolites of BCC and NST were compared. Statistical analyses of the levels were performed using commercial software (PASW, Version 17; SPSS Inc., Chicago, IL, USA; MedCalc, Mariakerke, Belgium). Student’s *t*-test was used for the comparison of the proportions. A P value lower than 0.05 was considered statistically significant. A receiver operation characteristic (ROC) curve was generated using a web server for metabolomics data analysis (MetaboAnalyst 3.0). [23]

## Results

### Baseline characteristics of patients and tissue samples

HR-MAS ^1^H NMR spectroscopic data were analyzed for all tissue samples, which included histopathologically confirmed 15 BCC tissues and 15 NST samples. The age of BCC patients ranged from 44 to 84 years with a mean of 67.3 ± 12.3 years. The patients consisted of four males (27%) and 11 females (73%). The histopathologic subtypes of BCC included nodular (n = 6), micronodular (n = 6), infiltrative (n = 2), and superficial (n = 1). NST samples were acquired from 10 BCC patients; additional five normal tissues were collected from patients with other cancers who underwent surgical treatment.

### Differences in metabolic profiling and univariate analysis

**Fig 1:**
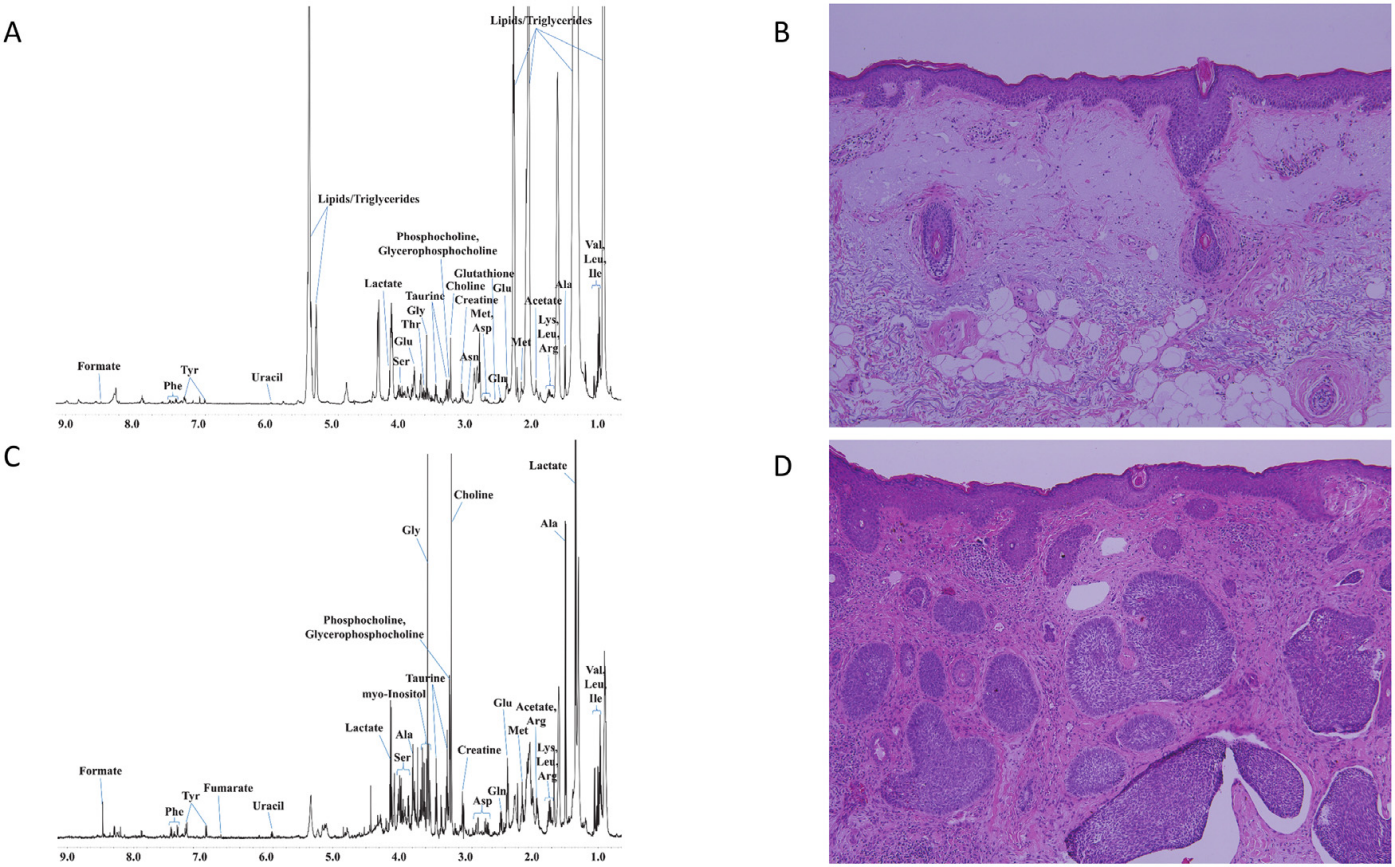
A representative 600 MHz ^1^H HR-MAS CPMG NMR spectra of normal skin (A) and basal cell carcinoma (C). Histopathologic images of normal skin (A) and basal cell carcinoma (D) (hematoxylin and eosin, original magnification X 100).

**Fig 2:**
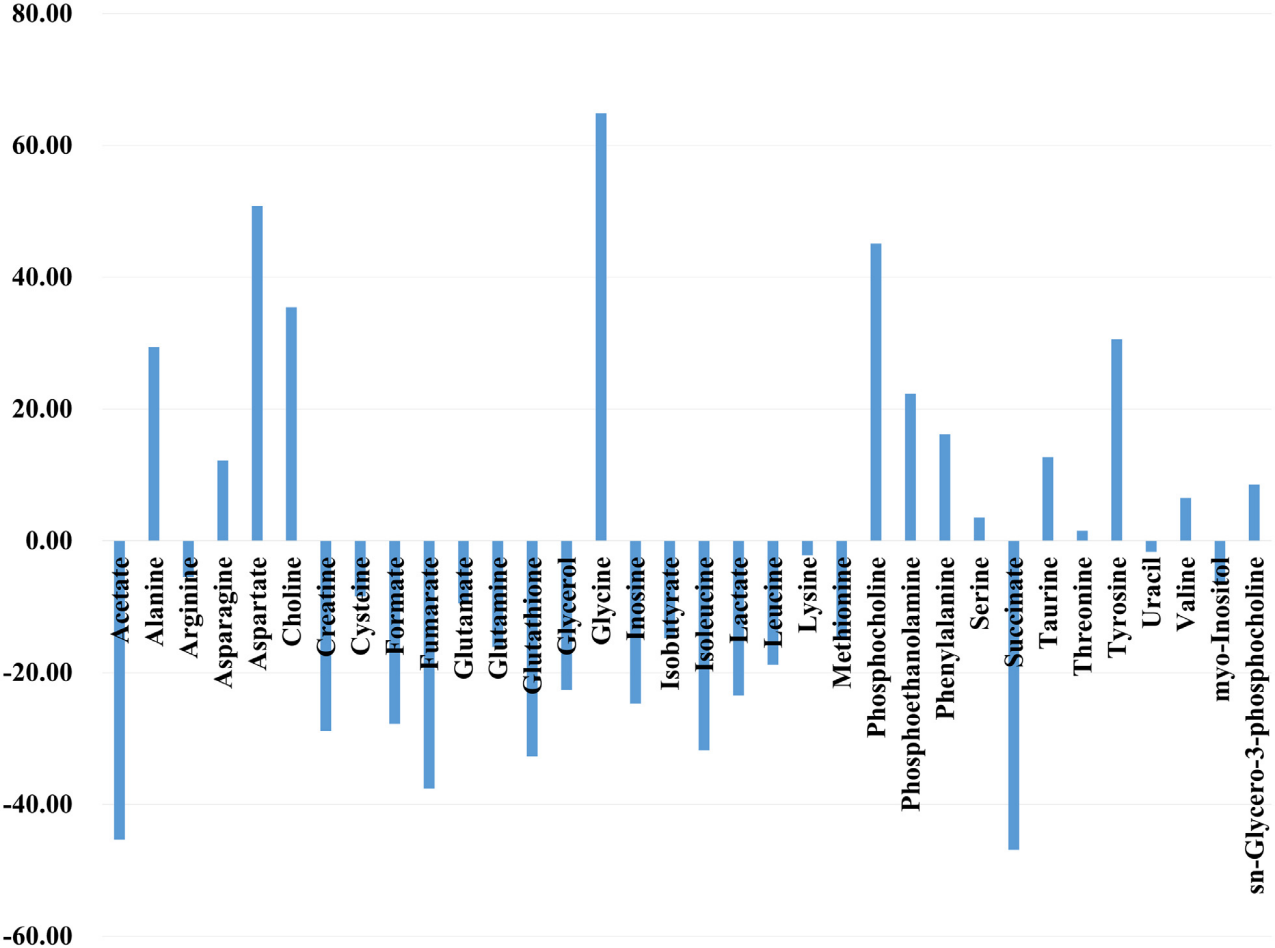
The percent change in metabolite levels in basal cell carcinoma tissues relative to the levels in normal tissues. % Change = ([basal cell carcinoma]–[normal])/[normal] × 100. The concentrations of the metabolites were calculated from the integration of peak areas using Chenomx.

The representative stacked HR-MAS ^1^H NMR spectra of BCC and NST are shown in Fig 1. Visual comparison of the spectra showed a variable degree of metabolites. In general, the level of lipid/triglyceride (TG) was higher in NST samples compared to the BCC samples. However, the exact level of lipid/TG was not compared because lipid/TG consist of several analog compounds. The mean quantities of marker metabolites used for the distinction of BCC and NST are shown in Table 1. The total areas of normalized spectra were used to calculate relative intensities of each metabolite presented as mean ± SEM. A selective non-overlapping resonance from each metabolite was used to calculate relative intensities. Among the marker metabolites examined for comparison of BCC and NST, the levels of 9 metabolites were significantly different. The levels of alanine (P = 0.005), aspartate (P = 0.011), glycine (P = 0.005), and phosphocholine (P = 0.021) were significantly elevated, and those of acetate (P = 0.008), creatine (P = 0.021), fumarate (P = 0.010), isoleucine (P < 0.001), and lactate (P = 0.016) decreased in BCC samples. Fig 2 presents the relative concentration of these metabolites in BCC and NST tissues.

**Table 1:** The relative concentrations of marker metabolites acquired by HR-MAS ^1^H NMR spectroscopy from basal cell carcinoma (BCC) and normal skin tissue (NST). Metabolites that were significantly different between tissues are shown in bold.

	BCC		NST		P values
	Mean	SE	Mean	SE	
Acetate	1.0268	0.1432	1.8791	0.2593	**0.008**
Alanine	8.7665	0.4817	6.7766	0.4471	**0.005**
Arginine	2.4139	0.1336	2.5541	0.1673	0.518
Asparagine	1.4250	0.1105	1.2705	0.1259	0.364
Aspartate	2.8929	0.2526	1.9180	0.2504	**0.011**
Choline	2.4864	0.3436	1.8362	0.1363	0.089
Creatine	0.9183	0.0938	1.2907	0.1205	**0.021**
Cysteine	0.9530	0.0948	1.0404	0.0910	0.511
Formate	1.0704	0.2917	1.4826	0.3052	0.337
Fumarate	0.0814	0.0101	0.1305	0.0144	**0.010**
Glutamate	7.2551	0.3589	8.0161	0.6114	0.292
Glutamine	2.2904	0.1428	2.7292	0.1716	0.059
Glutathione	0.2297	0.0305	0.3414	0.0875	0.238
Glycerol	2.4648	0.2406	3.1846	0.2705	0.057
Glycine	10.9154	0.5805	6.6196	0.3819	**<0.001**
Inosine	0.1623	0.0184	0.2156	0.0207	0.064
Isobutyrate	0.1186	0.0098	0.1391	0.0195	0.353
Isoleucine	1.4826	0.1227	2.1733	0.1183	**<0.001**
Lactate	17.5850	1.1913	22.9847	1.7356	**0.016**
Leucine	3.7121	0.2335	4.5711	0.3532	0.052
Lysine	1.9224	0.2278	1.9653	0.1956	0.887
Methionine	0.4716	0.0568	0.5554	0.0531	0.290
Phosphocholine	0.7126	0.0735	0.4911	0.0523	**0.021**
Phosphoethanolamine	2.7927	0.2385	2.2828	0.1364	0.074
Phenylalanine	1.3016	0.0967	1.1206	0.0870	0.175
Serine	7.8613	0.3669	7.5939	0.4621	0.654
Succinate	0.0941	0.0160	0.1771	0.0390	0.059
Taurine	5.1967	0.2901	4.6112	0.3319	0.195
Threonine	3.6117	0.2556	3.5584	0.2167	0.875
Tyrosine	1.1208	0.1013	0.8584	0.0807	0.052
Uracil	0.3693	0.0417	0.3754	0.0259	0.902
Valine	3.3027	0.1868	3.1011	0.2190	0.490
myo-Inositol	2.2990	0.2269	2.4508	0.1406	0.574
sn-Glycero-3-phosphocholine	1.7196	0.1681	1.5840	0.1129	0.509

### Multivariate analysis

**Fig 3:**
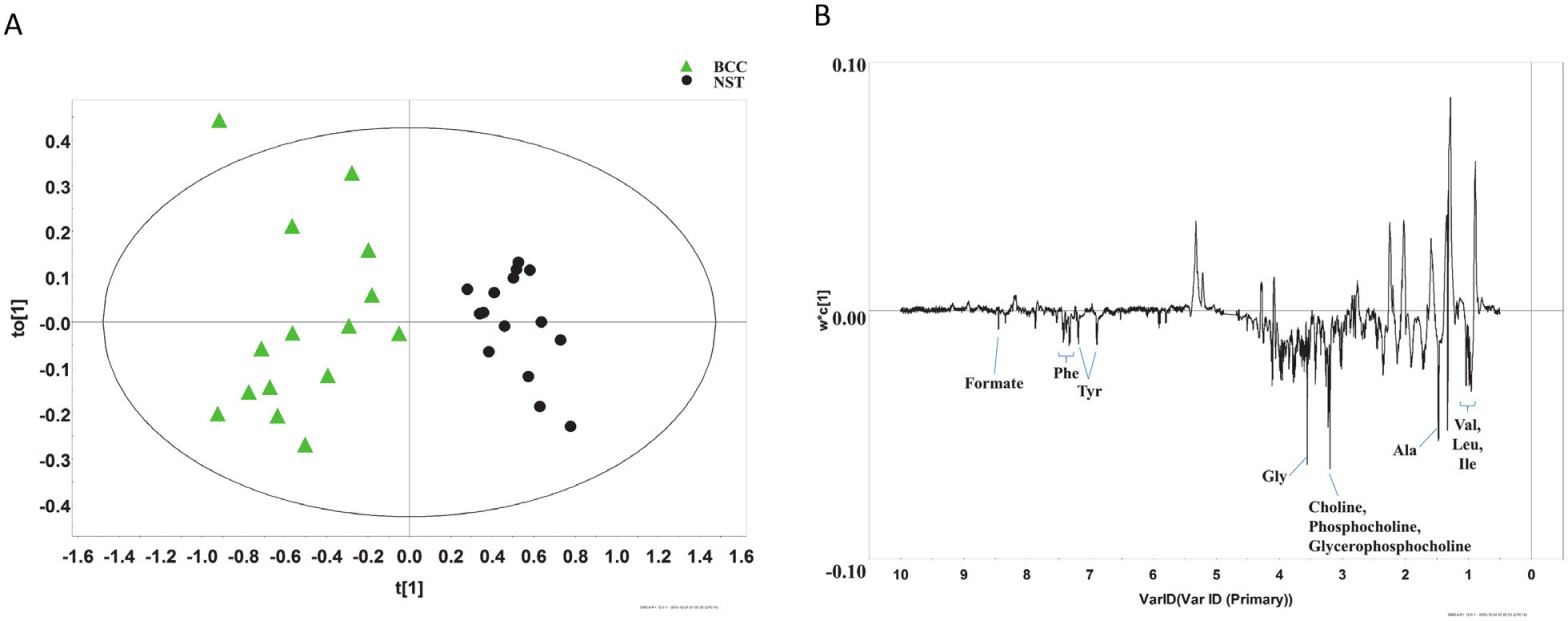
Multivariate analysis of ^1^H NMR spectra from basal cell carcinoma (BCC) and normal skin tissue (NST). (A) The OPLS-DA score plot clearly discriminates BCC from NST. (B) The corresponding loading plot.

**Fig 4:**
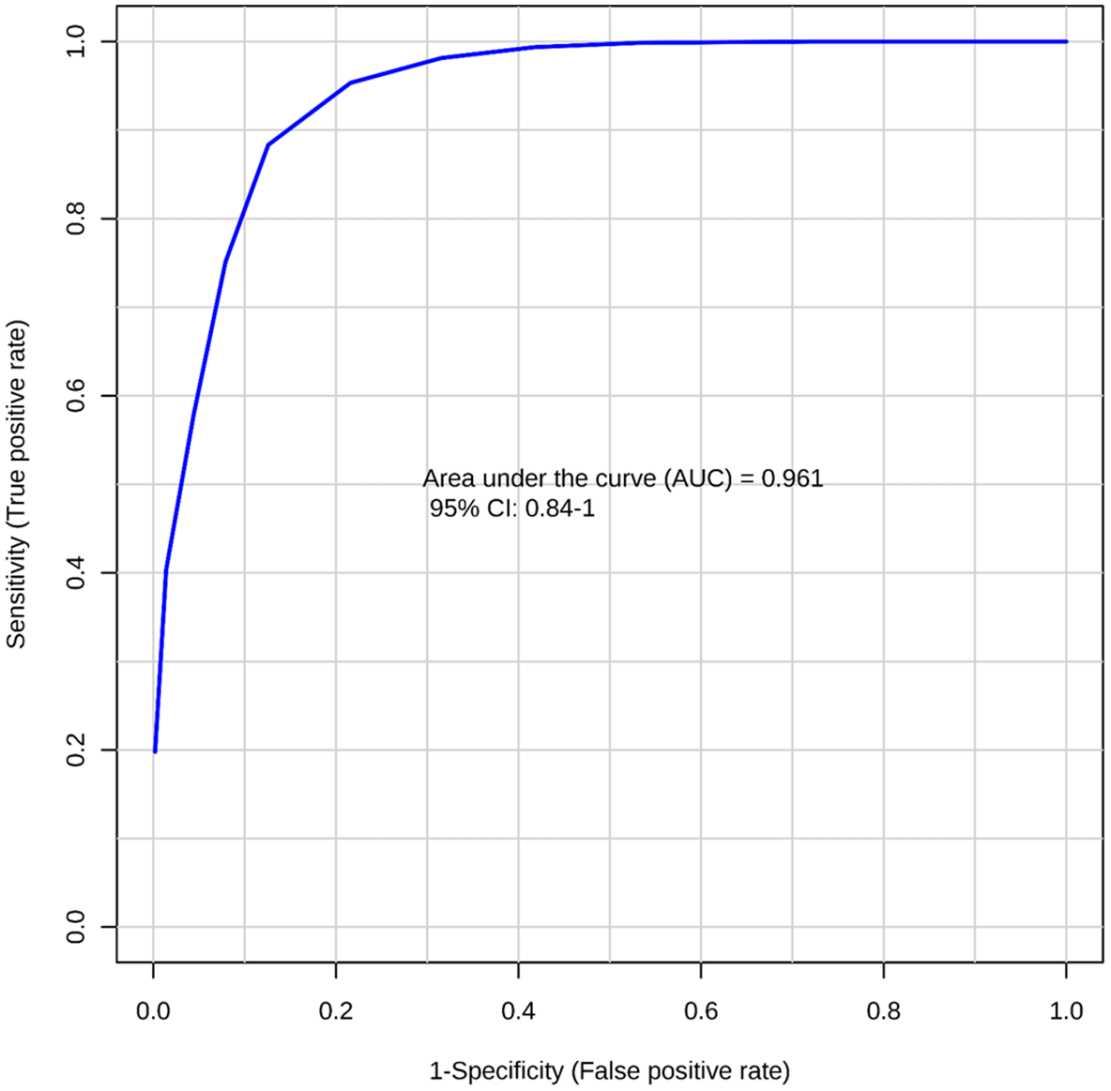
The receiver operator characteristic curve. Variables with significant prognostic value in the univariate analysis were included. An AUC of 0.961 represents excellent predictability in differentiating BCC from NST.

OPLS-DA score plot discriminated BCC from NST with a good description of the data (*R*^*2*^*X* = 0.784, *R*^*2*^*Y* = 0.855) and a good prediction accuracy for new data (*Q*^*2*^ = 0.679) (Fig 3). Metabolites with statistically significant differences in univariate analysis were included for the generation of ROC curves. ROC curves generated from the results revealed excellent discrimination of BCC from NST, which yielded an AUC value of 0.961 (Fig 4).

## Discussion

Recent advances in analytical biochemistry have led to the development of an array of metabolic profiling platforms with which to exploit the metabolome [24]. Recently, the application of HR-MAS NMR for the differentiation of the metabolic profiles of cancer and normal tissue has been explored for a variety of cancer types [25]. In the present study, we have applied HR-MAS ^1^H NMR spectroscopy combined with biostatistical methods to explore the metabolic profiling of BCC. To our knowledge, this is the first study to investigate the metabolite profile and metabolite intensity of BCC using HR-MAS ^1^H NMR.

The present study demonstrated that HR-MAS ^1^H NMR spectroscopy was able to discriminate BCC from normal skin tissue. The OPLS-DA models derived from the current metabolic analysis illustrates a clear separation between BCC and normal skin tissues, and thus demonstrates the potential of this statistical analytic approach in metabolomics. The univariate analysis found 9 metabolites that were significantly different between BCC and NST. The ROC curve generated from the results showed an excellent discrimination of BCC from NST (AUC value of 0.961).

The level of glycine was higher in BCC samples. Glycine is formed from the glycolytic intermediate 3-phosphoglycerate, and is an important source of one-carbon units for de novo purine synthesis [26]. Therefore, the increased levels of glycine could indicate enhanced nucleotide synthesis in BCC. Elevated glycine levels have been previously reported in other cancers, including breast, brain, ovarian, and colorectal cancer, in an MAS NMR study [13, 27–29].

The level of alanine was also higher in BCC samples. Alanine is a major source of energy for metabolism and a principal end-product of glycolysis/glutaminolysis in tumor proliferation and growth. Increased alanine levels were reported in other cancers such as prostatic and bladder cancers [12, 17]. Aspartate is a metabolite involved in the urea cycle and participates in gluconeogenesis. Therefore, the increased level of aspartate in BCC samples may be associated with gluconeogenesis.

Choline compounds are regarded as a marker of increased membrane turnover and are expected to increase in malignant tumors [30]. MRS techniques have detected alteration in the levels of choline-containing compounds in vivo in breast cancer patients and are now used for their diagnosis and prognosis [31]. The results of this study showed that the levels of phosphocholine were elevated in BCC. Therefore, the measurement of phosphocholine in patients with BCC by HR-MAS ^1^H NMR spectroscopy could be used as a biomarker for the diagnosis and prognosis of BCC patients.

Levels of acetate, creatine, fumarate, and isoleucine were lower in the BCC samples. We assume that the increased energy consumption of certain metabolites by the BCC cells may be associated with the decreased levels of these metabolites. In this study, lactate levels were also lower in the BCC samples. Previous studies have shown that increased lactate levels were detected in various cancer tissues [12, 25]. High concentrations of lactate in solid tumors have been associated with a high incidence of metastasis in the early stages of the disease, whereas lower concentrations of lactate indicate a longer and disease-free survival [32]. The low level of lactate in BCC may be attributed to the fact that BCC is a slowly growing, low-grade malignant tumor with rare metastatic potential.

The present study has some limitations. First, the results of this study are limited by a small sample size. Considering the many factors affecting metabolite changes in the tumor such as the histopathologic grade and heterogeneity of the tumor microenvironment, further studies with a larger sample size and different histopathologic subtypes are necessary to establish the metabolite profiles of BCC. Second, instead of analyzing fresh tissues, the tissues were preserved using the snap freezing technique, which could have affected the metabolite profile. Although some metabolic alteration is unavoidable, freezing the sample immediately after excision, minimizing the sample preparation time, and acquiring the MAS data at a low temperature can minimize degradation [15].

In conclusion, the HR-MAS ^1^H NMR-based metabolomic analysis showed a significant difference in the metabolic profiles of BCC and normal skin tissues. The results of this study suggest that HR-MAS ^1^H NMR spectroscopy can be a valuable tool in the diagnosis of BCC.

## Supporting Information

S1 Fig A representative 2D-NMR COSY spectrum of normal skin tissue. (TIF)
