# Supplementary figures and images for: Discrimination of Basal Cell Carcinoma from Normal Skin Tissue Using High-Resolution Magic Angle Spinning 1H NMR Spectroscopy
Source: PLoS One. 2016 Mar 2;11(3):e0150328. doi: 10.1371/journal.pone.0150328 (PMC4774902; doi:10.1371/journal.pone.0150328)

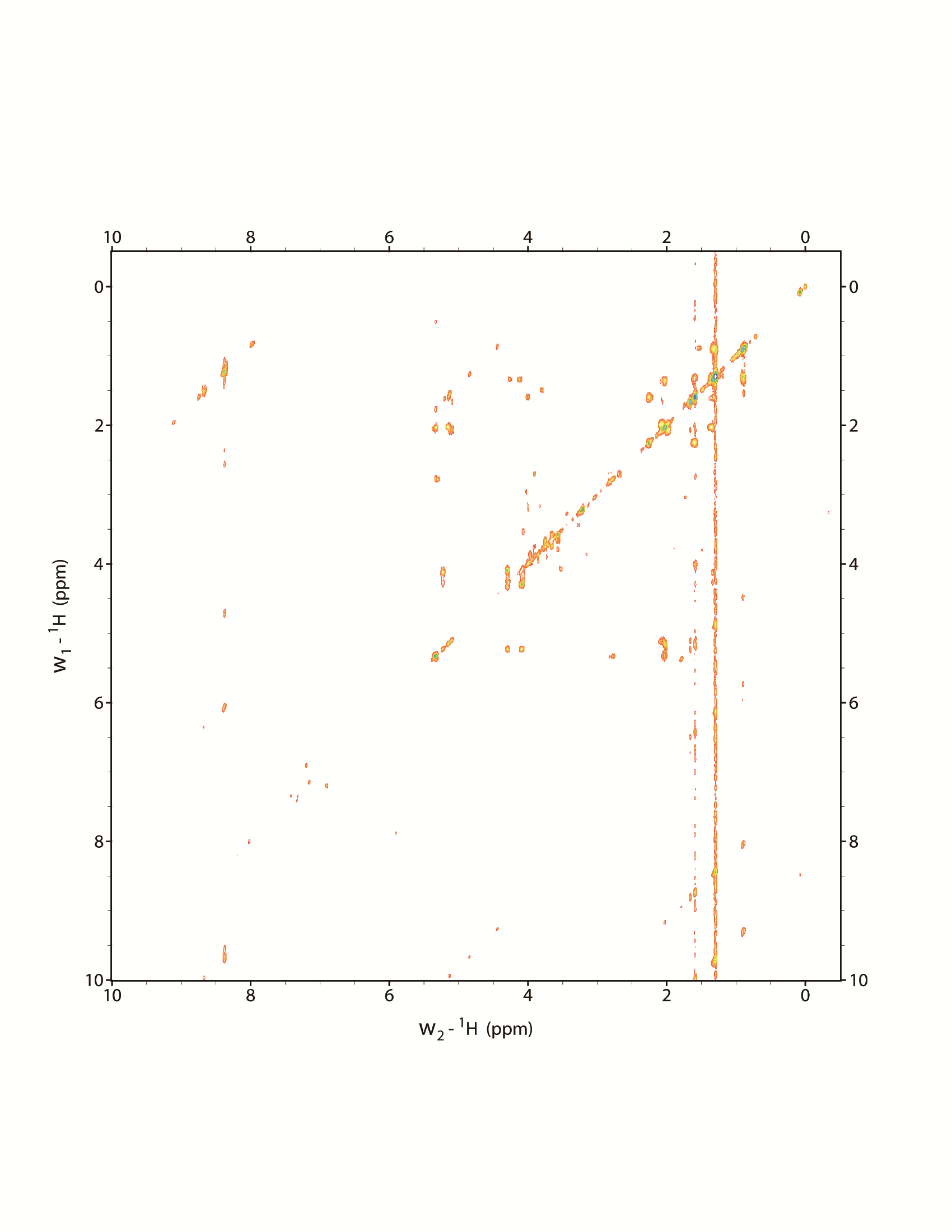

Supplement: S1 Fig — (TIF) [file pone.0150328.s001.tif]
